# Current Trends and Challenges in Drug-Likeness Prediction: Are They Generalizable and Interpretable?

**DOI:** 10.34133/hds.0098

**Published:** 2023-11-10

**Authors:** Wenyu Zhu, Yanxing Wang, Yan Niu, Liangren Zhang, Zhenming Liu

**Affiliations:** ^1^ State Key Laboratory of Natural and Biomimetic Drugs, School of Pharmaceutical Sciences, Peking University, 100191 Beijing, P. R. China.; ^2^ Department of Medicinal Chemistry, School of Pharmaceutical Sciences, Peking University, 100191 Beijing, P. R. China.

## Abstract

**Importance**: Drug-likeness of a compound is an overall assessment of its potential to succeed in clinical trials, and is essential for economizing research expenditures by filtering compounds with unfavorable properties and poor development potential. To this end, a robust drug-likeness prediction method is indispensable. Various approaches, including discriminative rules, statistical models, and machine learning models, have been developed to predict drug-likeness based on physiochemical properties and structural features. Notably, recent advancements in novel deep learning techniques have significantly advanced drug-likeness prediction, especially in classification performance.

**Highlights**: In this review, we addressed the evolving landscape of drug-likeness prediction, with emphasis on methods employing novel deep learning techniques, and highlighted the current challenges in drug-likeness prediction, specifically regarding the aspects of generalization and interpretability. Moreover, we explored potential remedies and outlined promising avenues for future research.

**Conclusion**: Despite the hurdles of generalization and interpretability, novel deep learning techniques have great potential in drug-likeness prediction and are worthy of further research efforts.

## Introduction

The lengthy drug development timelines and the low success rate in clinical trials give rise to significant risks and substantial expenses associated with bringing a drug to market [1–5]. It is advisable to filter out compounds with low development value in the early stage as tens of billions of them are tangible [6], and therefore to economize on wasteful expenditure in drug research and development.

Drug-likeness of a compound is defined by its physicochemical or structural similarity to a set of known drugs to holistically assess the potential for passing clinical trials. By screening chemical libraries with drug-likeness, compounds with potential adverse properties can be filtered out, and thereby reducing the risk in the later stages of drug development. A common category of drug-likeness evaluation methods is property-based rules defined as the acceptable thresholds on the physicochemical properties of drugs or investigational candidates [7–10], such as the well-known Lipinski's Rule of Five (RO5). Besides, drug-likeness can also be defined by the representative structural patterns of drugs [11–14].

However, simple rules based on properties and structural features might not cover the vast chemical space outside limited known drug or drug-like molecules. For better extrapolation capabilities, machine learning (ML) methods were introduced to model and predict drug-likeness, including artificial neural networks (ANNs) [15,16], support vector machines (SVMs) [17–22], and decision trees (DTs) [19,23,24]. Recently, with the resurgence of deep learning, many novel ANN methods were also employed to drug-likeness prediction [25–30].

While there are some publications [31–35] that have thoroughly reviewed drug-likeness prediction methods including rules and traditional ML models, and even from the perspective of ADME/T (absorption, distribution, metabolism, excretion, and toxicity), one that expatiates on methods using novel deep learning techniques is absent. In this review, we primarily focused on the assessment of drug-likeness based on methods derived directly from the set of known drugs, rather than on aspects such as ADME/T properties [36–41]. We overviewed drug-likeness prediction methods in a developmental manner and surveyed recent advances involving novel ANN techniques. From the two aspects of generalizability and interpretability, we proposed major challenges in drug-likeness research and discussed possible solutions and prospects.

### Drug-likeness filters and scorers defined on physicochemical properties and structural features

The earliest and most famous drug-likeness rule is RO5 [7], which is a group of 4 empirical rules summarized from clinical phase II drugs: (a) molecular weight ≤ 500, (b) octanol/water partition coefficient ≤ 5, (c) number of hydrogen bond donors ≤ 5, and (d) number of hydrogen bond acceptors ≤ 10. Compounds that meet 3 of these rules are considered as drug-like and well orally absorbed via passive transport. Thereafter, various empirical drug-likeness rules [8–10] have been developed based on the analysis of physicochemical properties of drugs or drug-like compound databases. Some studies [36–39] have also developed rules based on ADME/T properties in support of oral medications [42], considering their convenience and compliance.

**Fig. 1.**
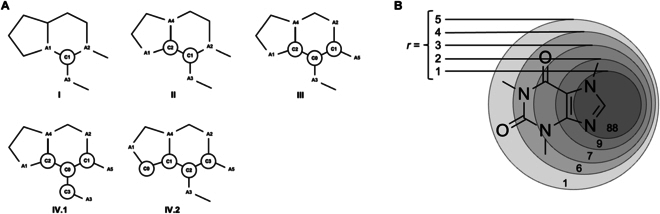
Drug-likeness methods defined on the comparison of substructure probabilities. (A) Multilevel chemical compatibility method extracts substructures by 5 kinds of atom-centered group labeled with letter C, and their side atoms labeled with letter A (adapted with permission from [12], Copyright 1999 American Chemical Society). (B) Substructures defined by Morgan algorithm includes topological structure centered in any atoms with radius *r*, and features of involved atoms (adapted with permission from [13], Copyright 2010 American Chemical Society).

Some analyses [43–46] of drug building blocks and frameworks in drug sets have found typical structural patterns that may help to understand drug-likeness concretely and to find the preferred frameworks and moieties for focused chemical library design. Therefore, drug-likeness methods can be defined by comparing the structural features of a compound to those of a set of known drugs from different perspectives. These include the distance to the cluster center extracted from drug building block descriptors [11], the comparison between the probability of substructures in the molecule query and in drugs [12,13] (Fig. 1), the presence of pharmacophores according to medicinal chemistry theory [14], and the principal components analysis in the space of physiochemical and structural descriptors [47].

Based on physiochemical and structural descriptors, optimization algorithms, such as genetic algorithm, were adapted to effectively explore the chemical space and obtain a diverse library with drug-like properties. SELECT [48], and its variant MoSELECT [49], which was combined with Pareto optimality to address multi-objection optimization, could be used to choose an optimal configuration for a multicomponent (e.g., amide) library. Genetic algorithm has also been used in drug-like feature selection for both substructure analysis [50] and ML modeling [18]. Also, search algorithms including Monte Carlo tree search were used to build a drug-like library with efficiency as well [51,52].

In addition to the binary discrimination between drug-like and non-drug-like molecules, a continuous measurement may be preferred for fine-grained assessment and flexible use. Quantitative estimate of drug-likeness (QED) [53] introduced a simple yet efficient method for multi-objective optimization, named desirability function, to provide a quantitative measurement of drug-likeness. Eight commonly used properties for drug-likeness assessment were taken into consideration, and desirability functions were obtained by fitting asymmetric double sigmoidal functions to these of oral drugs, respectively. The final QED score was given by (weighted) geometric averaging over desirability function values of the molecule:

$$\mathrm{QED}=\exp\left(\frac{\sum_{i=1}^{n}w_{i}\ln d_{i}}{\sum_{i=1}^{n}w_{i}}\right)$$

where *d_i_* indicates the *i*th desirability function with *w_i_* as its weight, and *n* is the number of properties being considered and equals to 8. Besides, continuous measurements also include the drug-likeness probability predictions of Bayesian probability theory [54], multivariate logistic regression [55], and various ML models.

There have been several proposals of new property indices recently to measure drug-likeness, such as the fraction lipophilicity index [56] and the fraction of sp^3^ carbon atoms [57]. These property-based rules are straightforward and easy to use, but they may not be adequate for dealing with more complex relationships between molecular features and the success rate in clinical trials. Methods based on structural features are conceptually consistent with fragment-based drug design and facilitate the construction of combinatorial chemistry-friendly design libraries. However, their main flaw lies in the overrestriction to the frameworks of known drugs or drug candidates, thus may potentially missing out on novel drug scaffolds in chemical space and limiting creativity in their application.

### Predicting drug-likeness with traditional ML models

Given the limited number of known drugs, ML methods were introduced to model and predict drug-likeness in consideration of their extrapolation capabilities. ML models are computer systems that learn and improve from data automatically [58]. Due to their ability to handle large-scale data and to separate complex features, ML models are widely used in various stages in drug development, providing board opportunities in research and innovation [59].

**Fig. 2.**
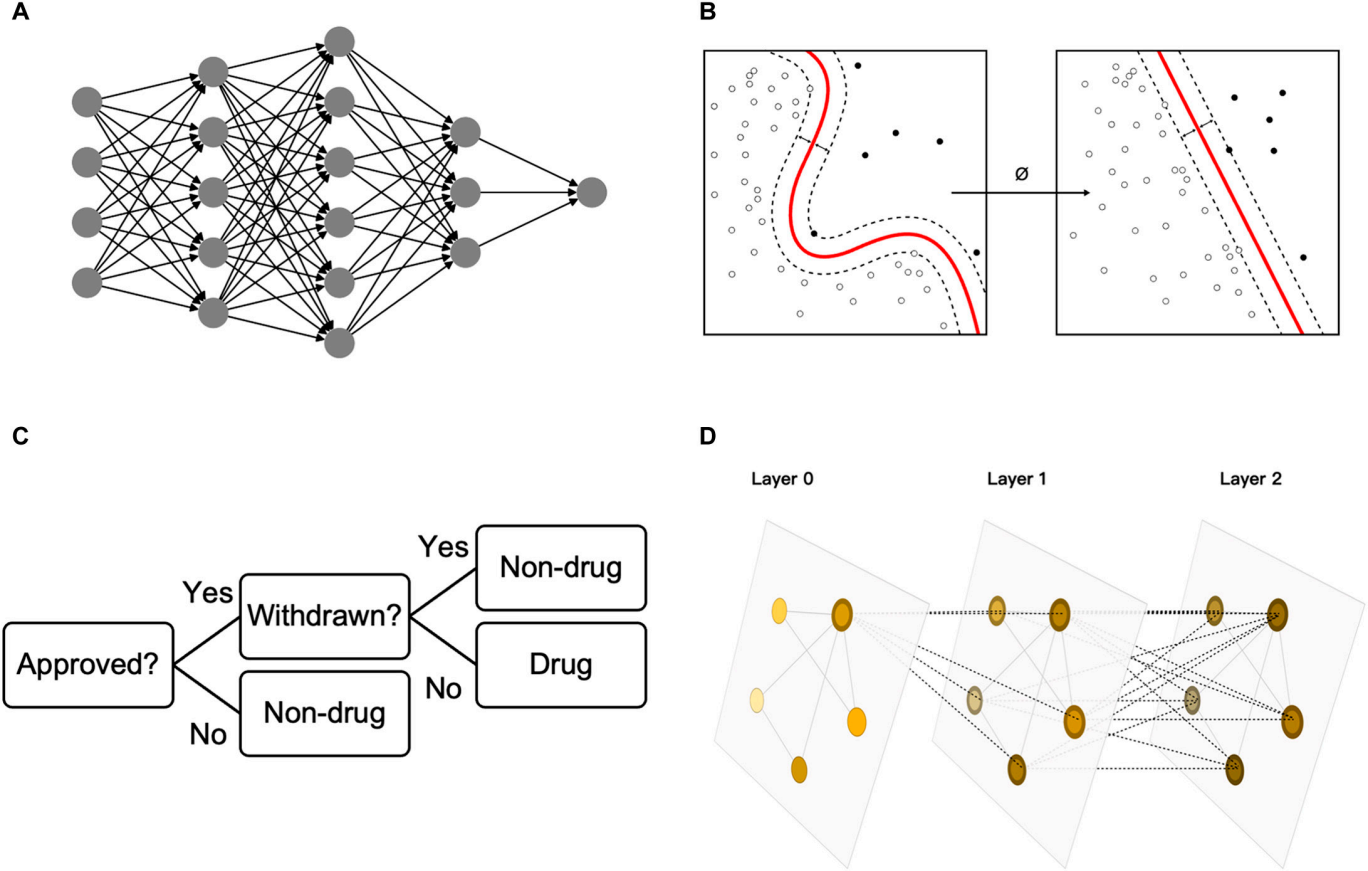
ML models used for drug-likeness prediction. (A) ANNs accept feature vectors as inputs, and output predictions through nonlinear transformations in the hidden layers. (B) Nonlinear kernels in SVM transform nonlinear separable data into linear separable space (https://en.wikipedia.org/wiki/Support_vector_machine#/media/File:Kernel_Machine.svg, CC BY-SA 4.0). (C) A diagram of DT deciding whether a compound is drug. (D) GNNs directly aggregate and process features from molecular graphs (adapted from [61], CC-BY 4.0).

ANNs (Fig. 2A) are a category of ML models that are characterized by nonlinear transformations and thus capable of fitting any function [60]. The use of ANNs to predict drug-likeness dates back to 1998, when Ajay and Murcko [15] used Bayesian neural networks (BNNs) to classify drug-like and non-drug-like molecules based on 7 molecular property descriptors and 166-bit MDL substructure fingerprints. In the same year, Sadowski and Kubinyi [16] used 92 Ghose–Crippen atomic types as descriptors and constructed a feedforward neural network to score the degree of drug-likeness for molecules. These early ANN methods outperformed property-based filters and even DT models in accuracy of discriminating between drug-like and non-drug-like structures. This predictive power was shown to generalize on external datasets.

SVM [62] (Fig. 2B) was introduced into research on drug-likeness prediction [17–22] as they became popular in the ML community. There is also a webserver, DrugMint [63], developed based on SVM. In comparative studies [17–19,64], SVM showed improvement in both performance and robustness, compared to ANN using the same descriptors. The performance of SVM in predicting drug-likeness is highly dependent on the input features. For instance, Li et al. [21] found that using a set of elaborately designed structural descriptors, extended connectivity fingerprints (ECFPs) [65], led to better accuracy than molecular properties or atom types (MOLPRINT 2D [66]). Moreover, Korkmaz et al. [22] improved the baseline SVM model by applying various features selection strategies. DT (Fig. 2C) was adopted in predicting drug-likeness as well. Though they exhibit similar or inferior performance to SVM [19], DT models have advantages in interpretability: Focusing on either moieties [23] or properties [24] depending on the initial condition settings, the branch conditions of DT models can be extracted and used as criteria for drug-like compound design.

Overall, most of them have achieved a modest classification performance of which accuracy reached 80%. Moreover, Li et al. [21] achieved 92.73% classification accuracy using ECFP4 and a larger dataset, which led to ~4% and ~5% raise, respectively.

### Drug-likeness prediction using novel deep learning models

**Fig. 3.**
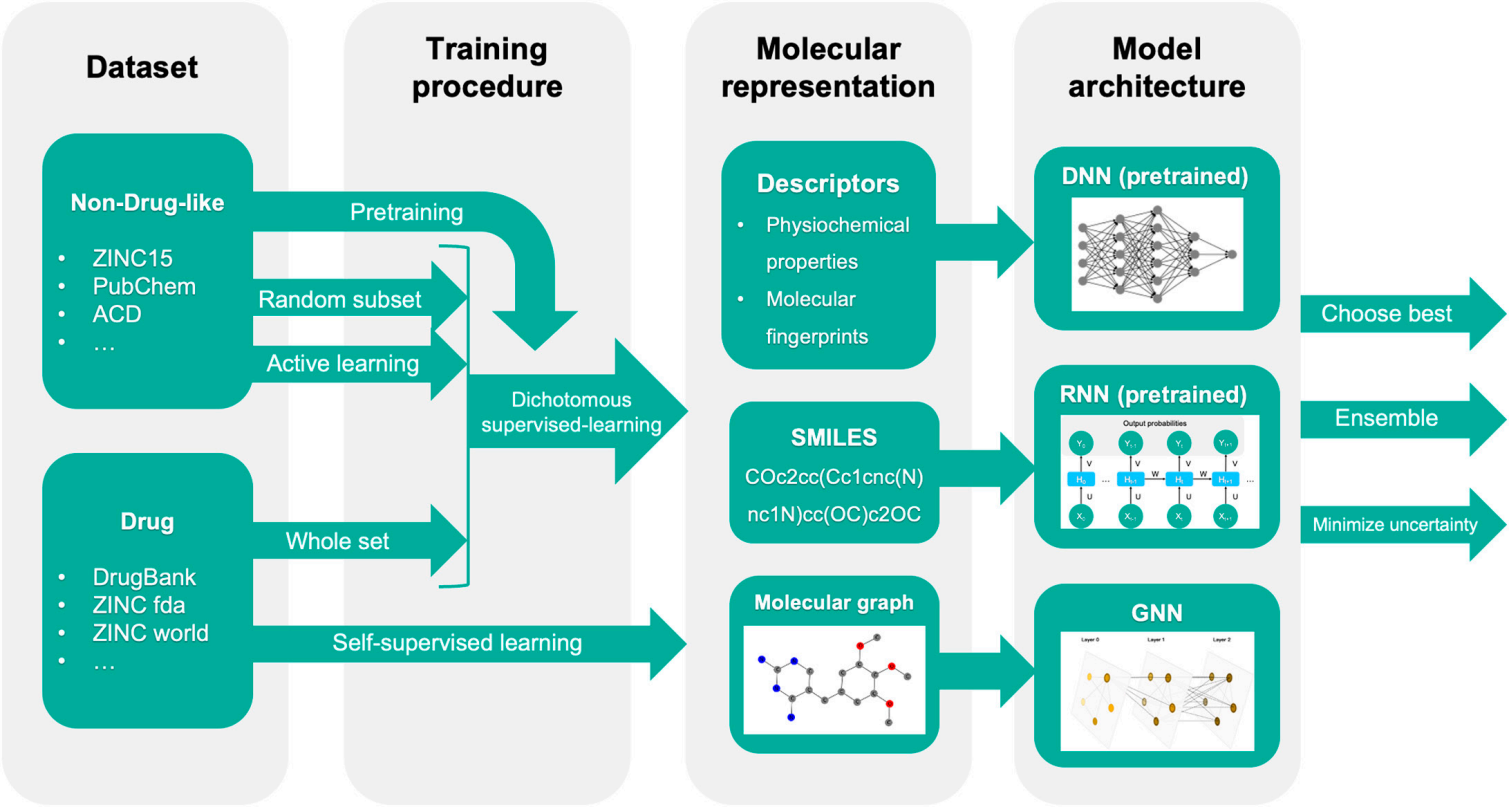
A sketch map for drug-likeness prediction methods based on novel ANN architectures mentioned in the “Drug-likeness Prediction Using Novel Deep Learning Models” section (GNN was adapted from [61], CC-BY 4.0).

With the resurgence of deep learning methods, drug-likeness prediction methods based on novel ANN architectures and molecular representations (as shown in Fig. 3) have been proposed for better classification performance.

Due to the relatively small data size of the drug set, pretraining seems a helpful strategy to leverage unlabeled molecules and learn broad chemical knowledge and thus improve the classification performance on the downstream drug-likeness prediction task in the later fine-tuning process. The work by Hu et al. [25], which used autoencoders (AEs) for pretraining, perhaps bridges traditional ANN methods and new ANN techniques. The predictive model was initialized with the parameters of the encoder part and then trained on classification task between drugs and non-drug-like molecules. Their model achieved better performance, which is 91% accuracy for drug-like/non-drug-like classification, and 97% for drug/non-drug-like, compared with early ANN and SVM methods. This may be attributed to basic chemical knowledge that the model learned during the pretraining process. Hooshmand et al. [26] also employed a pretraining approach to enhance the drug-likeness prediction using a deep belief network, where every 2 consecutive hidden layers make up a restricted Boltzmann machine and are pretrained layer-wise in a greedy manner using contrastive divergence algorithm. Their model achieved 97.75% accuracy on the leave-out test set, 2% better than Hu et al. [25], and 93.08% on the external test set, in which pretraining has contributed over 7% improvement.

Later studies are more concentrated on learning drug-likeness in an end-to-end way and use graph neural networks (GNNs; Fig. 2D) that directly operate on graphs built from molecular structures instead of elaborately designed descriptors. Beker et al. [27] evaluated multi-layer perceptron (MLP) classifiers with different descriptors as inputs, which were either randomly initialized or pretrained via an AE, as well as GNN with molecular graphs as inputs, and found minor gaps among their performance. The comparison suggests that the performance improvement compared to earlier studies is likely due to the use of more elaborate molecular representations rather than the model architectures. This is consistent with the satisfactory results achieved by Li et al. [21], who used SVM with ECFP rather than atom types or molecular descriptors as inputs. Inspired by the observation in some cases where one model made incorrect predictions with high variance while another’s were correct with low variance, they further improved the classification accuracy by combining 2 BNN modified from former models and retaining the less uncertain predictions. This adaptation raised the external accuracy of best predictors from 87 to 88% to 93%, which is the same level as Hooshmand et al. [26]. Sun et al. [28] predicted the drug-likeness by graph convolutional attention network (D-GCAN), which introduced an attention mechanism into GNN for drug-likeness prediction and achieved a 1% to 3% higher level of performance than the combined BNN of Beker et al. [27]. Cai et al. [29] developed a 3-subdivisional drug-likeness prediction model system, which consists of 3 individually trained models for evaluating the potential to reach in vivo, investigational, and approved stages progressively from in-stock compounds. They also combined active learning with ensemble learning to enhance the predictive ability of these models.

Whether to consider a non-drug-like set as negative background is a concern in drug-likeness prediction. Beker et al. [27] pointed out that QED is limited by its reliance on only the drugs and surveyed among 3 alternative non-drug datasets of the ZINC15 [67], the Network of Organic Chemistry (NOC) [68,69], and the Protein Data Bank (PDB) [70]. According to the evaluation results on the negative sets built through positive-unlabeled learning and the drug set, they observed that the drug/non-drug classifiers learned from ZINC15 showed better performance than others, and recommended ZINC15 as the negative set. On the contrary, Lee et al. [30] argued that these dichotomous models tend to learn ad hoc features discriminating between drugs and non-drug-like molecules rather than their features individually, by which their generalization is limited when non-drug-like molecules to be distinguished are substantially different from those in the negative training set. Instead, they adopted generative self-supervised learning to train a recurrent neural network (RNN) based on the SMILES strings of only drugs to fit their distribution. As the result, the drug-likeness scores of their self-supervised model showed relatively more consistent performance than the dichotomous GNN across different negative sets including GDB17 [71], ZINC15 [67], and ChEMBL [72], while the classification metric did drop 7% compared with the latter.

We concluded the aforementioned studies involving novel ANN techniques, as demonstrated in Table 1. Although differing in methods, these studies have primarily focused on developing classifiers between drugs and non-drug-like compounds based on their structures, and have demonstrated high predictive performance. We also listed drug/drug-like and non-drug-like databases in Table 2.

**Table 1.** Comparison of recent drug-likeness studies based on drug-likeness

Model category	Model	Molecular representation	Dataset	Performance		Description	Reference
				Accuracy	AUC ^a^		
MLP	AE	MOLD2 [73]	Drug: ZINC world;Drug-like: MDDR ^b^, WDI ^c^;Non-drug-like: ZINC, ACD ^d^	WDI/ACD = 91.04%MDDR/ZINC = 91.20%Drug/ZINC = 96.99%		Pretraining via autoencoding	[25]
MLP	Deep belief networks	Molecular fingerprints: MACCS Keys [74] (PubChem FP ^e^, ECFP)	Drug: ZINC world;Non-drug-like: ZINC	97%		Pretraining via contrastive divergence algorithm	[26]
MLP/GNN	Combined BNN (modified from MLP/AE/GNN)	RDKit ^f^, Mol2vec [75] (MOLD2, MCS ^g^, ECFP); Molecular graphs	Drug: DrugBank [76];Non-drug-like: ZINC, NOC, PDB	Base model = 87–88%Combined BNN = 93%		Retain less uncertain prediction from 2 BNN	[27]
GNN	D-GCAN	Molecular graphs	Drug: DrugBank;Non-drug-like: ZINC.	92.3%	0.951	Stack graph convolution and graph attention module	[28]
GNN	D-MPNN	Molecular graphs	Drug: ZINC world;Investigational: ZINC investigational;In vivo: ZINC in vivo;Non-drug-like: ZINC in stock.	In vivo/In stock = 94.8%Investigational/In vivo = 85.2%Drug/ Investigational = 82.2%		Build a system of 3 classifiers using active learning and ensemble	[29]
RNN	RNN with GRU	SMILES strings [77]	Drug: ZINC world-not-fda, ZINC fda;Non-drug-like: GDB17, ZINC, ChEMBL.		FDA/GDB17 = 0.979FDA/ZINC = 0.921FDA/ChEMBL = 0.824	Train only on the drug set using generative self-supervised learning	[30]

^a^ Area under receiver operating characteristic curve.

^b^ MDL Drug Data Report.

^c^ World Drug Index.

^d^ Available Chemical Directory.

^e^ Fingerprint encoding 881 structural features implemented in the PubChem database [78].

^f^ A set of 200 descriptors from the RDKit [79] library.

^g^ A bit vector denotes the presence or absence of 3,000 maximum common substructures appearing most frequently in the drug and non-drug datasets.

**Table 2.** Common-used databases for drug-likeness research

Category	Database		Size	Description	Website link	Freely available	Reference
Drug/drug-like	DrugBank		2,751/12,573	Well-annotated data of drugs approved by FDA, EMA, etc., and also investigational/experimental ones	https://go.drugbank.com/	Yes	[76]
	ZINC subsets	fda	1,379	FDA-approved drugs	https://zinc15.docking.org/substances/subsets/fda/	Yes	[67]
		world	3,447	Approved drugs in major juridications	https://zinc15.docking.org/substances/subsets/world/		
		in-trials	5,811	Compounds that have been investigated, including drugs	https://zinc15.docking.org/substances/subsets/in-trials/		
		in-man	98,168	Substances that have been in man	https://zinc15.docking.org/substances/subsets/in-man/		
		in-vivo	114,555	Substances tested in animals including man	https://zinc15.docking.org/substances/subsets/in-vivo/		
	MDL Drug Data Report (MDDR)		~180,000	Biologically relevant compounds with development phase annotated	https://www.3ds.com/products-services/biovia/	No		
	Comprehensive Medicinal Chemistry (CMC)		~8,000	Compounds used or studied as medicinal agents in humans	https://www.3ds.com/products-services/biovia/	No		
	World Drug Index (WDI)		~54,000	Data on marketed and development drugs worldwide	https://www.daylight.com/products/wdi.html	No		
Non-drug-like	ChEMBL		~2M	Compounds with bioassay data	https://www.ebi.ac.uk/chembl/	Yes	[72]
	ZINC15		~997M	Commercially available compounds	https://zinc15.docking.org/	Yes	[67]
	PubChem		~116M	Unique chemical structures extracted from contributed PubChem Substance records	https://pubchem.ncbi.nlm.nih.gov/	Yes	[78]
	GDB17		~166.4B (50M for download)	Small organic molecules from graph enumeration up to 17 atoms	https://gdb.unibe.ch/downloads/	Yes	[71]
	Available Chemicals Directory (ACD)		~12M	Commercially available chemicals	https://www.3ds.com/products-services/biovia/	No	

### Challenges and potential directions of drug-likeness prediction

Recent research on using ANN to predict drug-likeness may have gradually fallen into the trap of focusing solely on improving performance of dichotomous classification, neglecting the original purpose of drug-likeness, which is to exclude compounds with poor properties that are likely to fail in later stages. A desirable drug-likeness index should possess good generalizability and interpretability rather than pursuing classification performance all the way. The classification metrics directly demonstrate the ability to distinguish drugs from non-drug-like compounds in a mixed dataset, but the screening power of identifying the compounds with poor development value remains unclear. Drug-likeness research poses a unique challenge since, unlike tasks such as predicting solubility or activity, the only ground truth of drug-likeness to rely on is the results of clinical trials, which cost far more than laboratory tests. Therefore, it is nearly impossible to perform experimental ex post validation on a drug-likeness index.

The primary challenge in drug-likeness research is the demand for robust generalizability. Dichotomous models extract discriminating features between drugs and non-drug-like compounds, so the assigned probability is based on the ratio of distances between the given compound and drugs/non-drug-like molecules in the training set. However, it is impractical to construct an ideal negative dataset that encompass the entire chemical space of non-drug-like molecules, especially considering the data imbalance as there are only thousands of drugs. To address this challenge, some drug-likeness methods have employed subdivisional labels assigned by chemists [20] or incorporated research progress. [29] Some others only fit drugs to gain independence from the non-drug-like background, such as QED [53] and self-supervised RNN [30].

An emerging approach involves the utilization of multi-modal and large pretrained models, which have shown exceptional performance in fields such as natural language processing [80] and image generation [81]. For instance, transformers [82–84] pretrained on large-scale data [85,86], which were obtained from quantum chemistry calculations, have set new records in various molecular property prediction tasks [87,88]. Drug-likeness prediction would also benefit from molecular foundation models [89,90] for their general physiochemical and molecular structural knowledge learnt from pretraining. Yet, they are not well utilized.

Nevertheless, the scope of drugs is constantly changing with approvals and withdrawals by local drug administrations, rendering drug-likeness indices less effective over time. Drug discovery is inherently innovative, necessitating a robust drug-likeness prediction method that transcends the mere classification of compounds as drugs or non-drugs. It should possess the capability to identify compounds with desirable properties and high development potential in realistic scenarios that explore the vast chemical space.

Another significant challenge is the lack of interpretability, particularly in the context of ANN. These models are often referred to as “black boxes” due to their complex nonlinearity, which brings difficulty in understanding why and how they make predictions, despite their strong fitting capacity. Since direct ex post validation is impractical to perform, interpretability of drug-likeness methods becomes more important for validating from other perspectives, explaining why a molecule is “like a drug.” For example, if low prediction values of a drug-likeness method are generally attributed to some adverse properties like inferior pharmacokinetics or structural alerts indicating toxicity, we can rely on it more as an overall index that considers multiple factors. Besides, good interpretability could provide intuitive evidence to improve the confidence of medicinal chemists in the model, promoting cooperation between experimental research and drug-likeness prediction. Moreover, under the premise of good generalized performance, interpretable models could identify potential modifications that guide the design of new compounds with desirable properties.

Despite the significance of interpretability in ANN-based drug-likeness prediction, it has received little attention in recent studies. It is necessary to apply interpretation methods to these predictive models and construct compound datasets containing toxicity and other adverse properties for validating their interpretive performance. While gradient-based feature attribution [91,92] and subgraph recognition methods [93–96] could be employed handily on GNN-based drug-likeness models to attribute importance to structural features, other models, including MLP and RNN, may suffer from using molecular descriptors or SMILES strings that do not directly correspond to the molecular structure. Thus, when using ANN for drug-likeness prediction, it is important to carefully select the representation of input molecules. This can be achieved by incorporating medicinal chemistry knowledge into the model development process and utilizing feature engineering techniques to extract relevant atomic descriptors from the input data.

Last but not least, combining drug-likeness indices with one another and other methods could be a meaningful direction. Drug-likeness is associated with multifarious crucial properties for passing through clinical trials, and there is no direct regression label available for fitting. A single drug-likeness method is not capable of providing a comprehensive measurement. Combining multiple drug-likeness indices and other methods, leveraging their advantages, such as minimizing uncertainty, might be an effective way to obtain a more reliable drug-likeness index.

### Conclusion

The primary motivation behind this review was to address the evolving landscape of drug-likeness prediction, with emphasis on methods employing novel deep learning techniques. As novel ANN techniques develop, drug-likeness prediction methods have made great progress in classification accuracy, while also raising the risk of losing sight of the original purpose to filter compounds with adverse properties and poor development potential. Our aim was to highlight the challenges and potential solutions in this domain, and we focused on the need for both accuracy and the often-neglected aspects of generalizability and interpretability. As more attention is drawn on classifying drugs and non-drug-like molecules, their generalized performance remains unclear. There are several ways to tackle this, such as incorporating expert knowledge, employing large-scale pretraining, and improving non-drug-like set (or even becoming independent of it). Moreover, along with their strong capacity, ANNs have made drug-likeness prediction more opaque and thus less credible, which is an even more neglected problem that hinders the further practical use of drug-likeness and requires more efforts on exploring interpretation methods and built-in explainers. To sum up, in addition to accuracy, generalizability and interpretability are worthy of efforts, yet are neglected in drug-likeness research. A practicable way to develop reliable drug-likeness indices is to employ models that are more generalizable and interpretable, and combine them to obtain more robust and comprehensive performance.

## References

[1] DiMasi JA, Hansen RW, Grabowski HG. The price of innovation: New estimates of drug development costs. J Health Econ. 2003;22(2):151–185. 12606142 10.1016/S0167-6296(02)00126-1

[2] Adams CP, Brantner VV. Estimating the cost of new drug development: Is it really $802 million? Health Aff (Millwood). 2006;25(2):420–428. 16522582 10.1377/hlthaff.25.2.420

[3] DiMasi JA, Feldman L, Seckler A, Wilson A. Trends in risks associated with new drug development: Success rates for investigational drugs. Clin Pharmacol Ther. 2010;87(3):272–277. 20130567 10.1038/clpt.2009.295

[4] Hay M, Thomas DW, Craighead JL, Economides C, Rosenthal J. Clinical development success rates for investigational drugs. Nat Biotechnol. 2014;32(1):40–51. 24406927 10.1038/nbt.2786

[5] Wong CH, Siah KW, Lo AW. Estimation of clinical trial success rates and related parameters. Biostatistics. 2018;20(2):273–286. 10.1093/biostatistics/kxx069 PMC6409418 29394327

[6] Tingle BI, Tang KG, Castanon M, Gutierrez JJ, Khurelbaatar M, Dandarchuluun C, Moroz YS, Irwin JJ. ZINC-22—A free multi-billion-scale database of tangible compounds for ligand discovery. J Chem Inf Model. 2023;63(4):1166–1176. 36790087 10.1021/acs.jcim.2c01253 PMC9976280

[7] Lipinski CA, Lombardo F, Dominy BW, Feeney PJ. Experimental and computational approaches to estimate solubility and permeability in drug discovery and development settings. Adv Drug Del Rev. 1997;23(1):3–25. 10.1016/s0169-409x(00)00129-0 11259830

[8] Ghose AK, Viswanadhan VN, Wendoloski JJ. A knowledge-based approach in designing combinatorial or medicinal chemistry libraries for drug discovery. 1. A qualitative and quantitative characterization of known drug databases. J Comb Chem. 1999;1(1):55–68. 10746014 10.1021/cc9800071

[9] Oprea TI. Property distribution of drug-related chemical databases. J Comput Aided Mol Des. 2000;14(3):251–264. 10756480 10.1023/a:1008130001697

[10] Zheng S, Luo X, Chen G, Zhu W, Shen J, Chen K, Jiang H. A new rapid and effective chemistry space filter in recognizing a druglike database. J Chem Inf Model. 2005;45(4):856–862. 16045278 10.1021/ci050031j

[11] Xu J, Stevenson J. Drug-like Index: A new approach to measure drug-like compounds and their diversity. J Chem Inf Comput Sci. 2000;40(5):1177–1187. 11045811 10.1021/ci000026+

[12] Wang J, Ramnarayan K. Toward designing drug-like libraries: A novel computational approach for prediction of drug feasibility of compounds. J Comb Chem. 1999;1(6):524–533. 10748729 10.1021/cc990032m

[13] Ursu O, Oprea TI. Model-free drug-likeness from fragments. J Chem Inf Model. 2010;50(8):1387–1394. 20726597 10.1021/ci100202p

[14] Muegge I, Heald SL, Brittelli D. Simple selection criteria for drug-like chemical matter. J Med Chem. 2001;44(12):1841–1846. 11384230 10.1021/jm015507e

[15] Ajay WP, Murcko MA. Can we learn to distinguish between “drug-like” and “nondrug-like” molecules? J Med Chem. 1998;41(18):3314–3324. 9719583 10.1021/jm970666c

[16] Sadowski J, Kubinyi H. A scoring scheme for discriminating between drugs and nondrugs. J Med Chem. 1998;41(18):3325–3329. 9719584 10.1021/jm9706776

[17] Byvatov E, Fechner U, Sadowski J, Schneider G. Comparison of support vector machine and artificial neural network systems for drug/nondrug classification. J Chem Inf Comput Sci. 2003;43(6):1882–1889. 14632437 10.1021/ci0341161

[18] Zernov VV, Balakin KV, Ivaschenko AA, Savchuk NP, Pletnev IV. Drug discovery using support vector machines. The case studies of drug-likeness, agrochemical-likeness, and enzyme inhibition predictions. J Chem Inf Comput Sci. 2003;43(6):2048–2056. 14632457 10.1021/ci0340916

[19] Müller K-R, Rätsch G, Sonnenburg S, Mika S, Grimm M, Heinrich N. Classifying ‘drug-likeness’ with kernel-based learning methods. J Chem Inf Model. 2005;45(2):249–253. 15807485 10.1021/ci049737o

[20] Takaoka Y, Endo Y, Yamanobe S, Kakinuma H, Okubo T, Shimazaki Y, Ota T, Sumiya S, Yoshikawa K. Development of a method for evaluating drug-likeness and ease of synthesis using a data set in which compounds are assigned scores based on chemists’ intuition. J Chem Inf Comput Sci. 2003;43(4):1269–1275. 12870920 10.1021/ci034043l

[21] Li Q, Bender A, Pei J, Lai L. A large descriptor set and a probabilistic kernel-based classifier significantly improve druglikeness classification. J Chem Inf Model. 2007;47(5):1776–1786. 17718552 10.1021/ci700107y

[22] Korkmaz S, Zararsiz G, Goksuluk D. Drug/nondrug classification using support vector machines with various feature selection strategies. Comput Methods Prog Biomed. 2014;117(2):51–60. 10.1016/j.cmpb.2014.08.009 25224081

[23] Wagener M, van Geerestein VJ. Potential drugs and nondrugs: Prediction and identification of important structural features. J Chem Inf Comput Sci. 2000;40(2):280–292. 10761129 10.1021/ci990266t

[24] Schneider N, Jäckels C, Andres C, Hutter MC. Gradual in silico filtering for druglike substances. J Chem Inf Model. 2008;48(3):613–628. 18269264 10.1021/ci700351y

[25] Hu Q, Feng M, Lai L, Pei J. Prediction of drug-likeness using deep autoencoder neural networks. Front Genet. 2018;9, 1. 30538725 10.3389/fgene.2018.00585 PMC6277570

[26] Hooshmand SA, Jamalkandi SA, Alavi SM, Masoudi-Nejad A. Distinguishing drug/non-drug-like small molecules in drug discovery using deep belief network. Mol Divers. 2021;25(2):827–838. 32193758 10.1007/s11030-020-10065-7

[27] Beker W, Wołos A, Szymkuć S, Grzybowski BA. Minimal-uncertainty prediction of general drug-likeness based on Bayesian neural networks. Nat Mach Intell. 2020;2(8):457–465.

[28] Sun J, Wen M, Wang H, Ruan Y, Yang Q, Kang X, Zhang H, Zhang Z, Lu H. Prediction of drug-likeness using graph convolutional attention network. Bioinformatics. 2022;38(23):5262–5269. 36222555 10.1093/bioinformatics/btac676

[29] Cai C, Lin H, Wang H, Xu Y, Ouyang Q, Lai L, Pei J. MiDruglikeness: Subdivisional drug-likeness prediction models using active ensemble learning strategies. Biomol Ther. 2023;13(1):29. 10.3390/biom13010029 PMC9855665 36671415

[30] Lee K, Jang J, Seo S, Lim J, Kim WY. Drug-likeness scoring based on unsupervised learning. Chem Sci. 2022;13(2):554–565. 35126987 10.1039/d1sc05248a PMC8729801

[31] Clark DE, Pickett SD. Computational methods for the prediction of ‘drug-likeness. Drug Discov Today. 2000;5(2):49–58. 10.1016/s1359-6446(99)01451-8 10652455

[32] Walters WP, Murcko MA. Prediction of ‘drug-likeness’. Adv Drug Del Rev. 2002;54(3):255–271. 10.1016/s0169-409x(02)00003-0 11922947

[33] Tian S, Wang J, Li Y, Li D, Xu L, Hou T. The application of in silico drug-likeness predictions in pharmaceutical research. Adv Drug Del Rev. 2015;86:2–10. 10.1016/j.addr.2015.01.009 25666163

[34] Agoni C, Olotu FA, Ramharack P, Soliman ME. Druggability and drug-likeness concepts in drug design: Are biomodelling and predictive tools having their say? J Mol Model. 2020;26(6):120. 32382800 10.1007/s00894-020-04385-6

[35] Jia C-Y, Li J-Y, Hao G-F, Yang G-F. A drug-likeness toolbox facilitates ADMET study in drug discovery. Drug Discov Today. 2020;25(1):248–258. 31705979 10.1016/j.drudis.2019.10.014

[36] Veber DF, Johnson SR, Cheng H-Y, Smith BR, Ward KW, Kopple KD. Molecular properties that influence the oral bioavailability of drug candidates. J Med Chem. 2002;45(12):2615–2623. 12036371 10.1021/jm020017n

[37] Martin YC. A bioavailability score. J Med Chem. 2005;48(9):3164–3170. 15857122 10.1021/jm0492002

[38] Johnson TW, Dress KR, Edwards M. Using the golden triangle to optimize clearance and oral absorption. Bioorg Med Chem Lett. 2009;19(19):5560–5564. 19720530 10.1016/j.bmcl.2009.08.045

[39] Waring MJ. Defining optimum lipophilicity and molecular weight ranges for drug candidates—Molecular weight dependent lower LogD limits based on permeability. Bioorg Med Chem Lett. 2009;19(10):2844–2851. 19361989 10.1016/j.bmcl.2009.03.109

[40] Daina A, Michielin O, Zoete V. SwissADME: A free web tool to evaluate pharmacokinetics, drug-likeness and medicinal chemistry friendliness of small molecules. Sci Rep. 2017;7(1):42717. 28256516 10.1038/srep42717 PMC5335600

[41] Xiong G, Wu Z, Yi J, Fu L, Yang Z, Hsieh C, Yin M, Zeng X, Wu C, Lu A, et al. ADMETlab 2.0: An integrated online platform for accurate and comprehensive predictions of ADMET properties. Nucleic Acids Res. 2021;49(W1):W5–W14. 33893803 10.1093/nar/gkab255 PMC8262709

[42] Shahiwala A. Formulation approaches in enhancement of patient compliance to oral drug therapy. Expert Opin Drug Deliv. 2011;8(11):1521–1529. 21995544 10.1517/17425247.2011.628311

[43] Bemis GW, Murcko MA. The properties of known drugs. 1. Molecular frameworks. J Med Chem. 1996;39(15):2887–2893. 8709122 10.1021/jm9602928

[44] Bemis GW, Murcko MA. Properties of known drugs. 2. Side chains. J Med Chem. 1999;42(25):5095–5099. 10602694 10.1021/jm9903996

[45] Siegel MG, Vieth M. Drugs in other drugs: A new look at drugs as fragments. Drug Discov Today. 2007;12(1):71–79. 17198975 10.1016/j.drudis.2006.11.011

[46] Wang J, Hou T. Drug and drug candidate building block analysis. J Chem Inf Model. 2010;50(1):55–67. 20020714 10.1021/ci900398f

[47] García-Sosa AT, Oja M, Hetényi C, Maran U. Disease-specific differentiation between drugs and non-drugs using principal component analysis of their molecular descriptor space. Mol Inform. 2012;31(5):369–383. 27477266 10.1002/minf.201100094

[48] Gillet VJ, Willett P, Bradshaw J, Green DVS. Selecting combinatorial libraries to optimize diversity and physical properties. J Chem Inf Comput Sci. 1999;39(1):169–177.

[49] Gillet VJ, Khatib W, Willett P, Fleming PJ, Green DVS. Combinatorial library design using a multiobjective genetic algorithm. J Chem Inf Comput Sci. 2002;42(2):375–385. 11911707 10.1021/ci010375j

[50] Gillet VJ, Willett P, Bradshaw J. Identification of biological activity profiles using substructural analysis and genetic algorithms. J Chem Inf Comput Sci. 1998;38(2):165–179. 9538517 10.1021/ci970431+

[51] Brown RD, Hassan M, Waldman M. Combinatorial library design for diversity, cost efficiency, and drug-like character. J Mol Graph Model. 2000;18(4):427–437. 11143560 10.1016/s1093-3263(00)00072-3

[52] Pickett SD, McLay IM, Clark DE. Enhancing the hit-to-lead properties of lead optimization libraries. J Chem Inf Comput Sci. 2000;40(2):263–272. 10761127 10.1021/ci990261w

[53] Bickerton GR, Paolini GV, Besnard J, Muresan S, Hopkins AL. Quantifying the chemical beauty of drugs. Nat Chem. 2012;4(2):90–98. 22270643 10.1038/nchem.1243 PMC3524573

[54] Yusof I, Segall MD. Considering the impact drug-like properties have on the chance of success. Drug Discov Today. 2013;18(13):659–666. 23458995 10.1016/j.drudis.2013.02.008

[55] García-Sosa AT, Oja M, Hetényi C, Maran U. DrugLogit: Logistic discrimination between drugs and nondrugs including disease-specificity by assigning probabilities based on molecular properties. J Chem Inf Model. 2012;52(8):2165–2180. 22830445 10.1021/ci200587h

[56] Tsantili-Kakoulidou A, Demopoulos VJ. Fraction lipophilicity index (FLI). A drug-like metric for orally administered ionizable drugs. SAR QSAR Environ Res. 2019;30(9):643–653. 31469319 10.1080/1062936X.2019.1653363

[57] Wei W, Cherukupalli S, Jing L, Liu X, Zhan P. Fsp3: A new parameter for drug-likeness. Drug Discov Today. 2020;25(10):1839–1845. 32712310 10.1016/j.drudis.2020.07.017

[58] Jordan MI, Mitchell TM. Machine learning: Trends, perspectives, and prospects. Science. 2015;349(6245):255–260. 26185243 10.1126/science.aaa8415

[59] Vamathevan J, Clark D, Czodrowski P, Dunham I, Ferran E, Lee G, Li B, Madabhushi A, Shah P, Spitzer M, et al. Applications of machine learning in drug discovery and development. Nat Rev Drug Discov. 2019;18(6):463–477. 30976107 10.1038/s41573-019-0024-5 PMC6552674

[60] Cybenko G. Approximation by superpositions of a sigmoidal function. Math Control Signals Syst. 1989;2(4):303–314.

[61] Sanchez-Lengeling B, Reif E, Pearce A, Wiltschko AB. A gentle introduction to graph neural networks. Distill. 2021;6(9): Article e33.

[62] Cortes C, Vapnik V. Support-vector networks. Mach Learn. 1995;20(3):273–297.

[63] Dhanda SK, Singla D, Mondal AK, Raghava GP. DrugMint: A webserver for predicting and designing of drug-like molecules. Biol Direct. 2013;8(1):28. 24188205 10.1186/1745-6150-8-28 PMC3826839

[64] Tang K, Zhu R, Li Y, Cao Z. Discrimination of approved drugs from experimental drugs by learning methods. BMC Bioinformatics. 2011;12(1):157. 21569562 10.1186/1471-2105-12-157 PMC3120701

[65] Rogers D, Hahn M. Extended-connectivity fingerprints. J Chem Inf Model. 2010;50(5):742–754. 20426451 10.1021/ci100050t

[66] Bender A, Mussa HY, Glen RC, Reiling S. Molecular similarity searching using atom environments, information-based feature selection, and a naïve Bayesian classifier. J Chem Inf Comput Sci. 2004;44(1):170–178. 14741025 10.1021/ci034207y

[67] Sterling T, Irwin JJ. ZINC 15—Ligand discovery for everyone. J Chem Inf Model. 2015;55(11):2324–2337. 26479676 10.1021/acs.jcim.5b00559 PMC4658288

[68] Fialkowski M, Bishop KJM, Chubukov VA, Campbell CJ, Grzybowski BA. Architecture and evolution of organic chemistry. Angew Chem Int Ed. 2005;44(44):7263–7269. 10.1002/anie.200502272 16276556

[69] Kowalik M, Gothard CM, Drews AM, Gothard NA, Weckiewicz A, Fuller PE, Grzybowski BA, Bishop KJM. Parallel optimization of synthetic pathways within the network of organic chemistry. Angew Chem. 2012;124(32):8052–8056. 10.1002/anie.201202209 22807100

[70] Berman H, Henrick K, Nakamura H. Announcing the worldwide protein data bank. Nat Struct Mol Biol. 2003;10(12):980–980. 10.1038/nsb1203-980 14634627

[71] Ruddigkeit L, Van Deursen R, Blum LC, Reymond J-L. Enumeration of 166 billion organic small molecules in the chemical universe database GDB-17. J Chem Inf Model. 2012;52(11):2864–2875. 23088335 10.1021/ci300415d

[72] Mendez D, Gaulton A, Bento AP, Chambers J, De Veij M, Félix E, Magariños MP, Mosquera JF, Mutowo P, Nowotka M, et al. ChEMBL: Towards direct deposition of bioassay data. Nucleic Acids Res. 2018;47(D1):D930–D940. 10.1093/nar/gky1075 PMC6323927 30398643

[73] Hong H, Xie Q, Ge W, Qian F, Fang H, Shi L, Su Z, Perkins R, Tong W. Mold2, molecular descriptors from 2D structures for chemoinformatics and toxicoinformatics. J Chem Inf Model. 2008;48(7):1337–1344. 18564836 10.1021/ci800038f

[74] Durant JL, Leland BA, Henry DR, Nourse JG. Reoptimization of MDL keys for use in drug discovery. J Chem Inf Comput Sci. 2002;42(6):1273–1280. 12444722 10.1021/ci010132r

[75] Jaeger S, Fulle S, Turk S. Mol2vec: Unsupervised machine learning approach with chemical intuition. J Chem Inf Model. 2018;58(1):27–35. 29268609 10.1021/acs.jcim.7b00616

[76] Wishart DS, Feunang YD, Guo AC, Lo EJ, Marcu A, Grant JR, Sajed T, Johnson D, Li C, Sayeeda Z, et al. DrugBank 5.0: A major update to the DrugBank database for 2018. Nucleic Acids Res. 2018;46(D1):D1074–D1082. 29126136 10.1093/nar/gkx1037 PMC5753335

[77] Weininger DSMILES, a Chemical Language and Information System. 1. Introduction to methodology and encoding rules. J Chem Inf Comput Sci. 1988;28(1):31–36.

[78] Bolton EE, Wang Y, Thiessen PA, Bryant, S. H. Chapter 12 - PubChem: Integrated platform of small molecules and biological activities. In: Wheeler RA, Spellmeyer DC, editors. *Annual Reports in Computational Chemistry*. Amsterdam (Netherlands): Elsevier; 2008. p. 217–241.

[79] Landrum G. RDKit: A software suite for cheminformatics, computational chemistry, and predictive modeling. Greg Landrum. 2013;8:1.

[80] Brown TB, Mann B, Ryder N, Subbiah M, Kaplan J, Dhariwal P, Neelakantan A, Shyam P, Sastry G, Askell A, et al. Amodei. D language models are few-shot learners. arXiv. July 2020;22.

[81] Ramesh A, Dhariwal P, Nichol A, Chu C. Chen, M. Hierarchical text-conditional image generation with CLIP latents. arXiv. April 2022;12.

[82] Rong Y, Bian Y, Xu T, Xie W, WEI Y, Huang W, Huang J. Self-supervised graph transformer on large-scale molecular data. Paper presented at: Advances in Neural Information Processing Systems. Curran Associates Inc. 2020;33:12559–12571.

[83] Ying C, Cai T, Luo S, Zheng S, Ke G, He D, Shen Y, Liu T-Y. Do transformers really perform badly for graph representation?, Paper presented at: Advances in Neural Information Processing Systems. Curran Associates Inc. 2021;34:28877–28888.

[84] Zhou G, Gao Z, Ding Q, Zheng H, Xu H, Wei Z, Zhang L, Ke G.Uni-Mol: A universal 3D molecular representation learning framework. ChemRxiv. March 7, 2023.

[85] Ramakrishnan R, Dral PO, Rupp M, von Lilienfeld OA. Quantum chemistry structures and properties of 134 kilo molecules. Sci Data. 2014;1(1): Article 140022. 25977779 10.1038/sdata.2014.22 PMC4322582

[86] Hu W, Fey M, Ren H, Nakata M, Dong Y. Leskovec. J OGB-LSC: A large-scale challenge for machine learning on graphs. arXiv. October 2021;20.

[87] Wu Z, Ramsundar B, Feinberg N, Gomes J, Geniesse C, Pappu AS, Leswing K, Pande V, Gomes E, Geniesse J, et al. MoleculeNet: A benchmark for molecular machine learning. Chem Sci. 2018;9(2):513–530. 29629118 10.1039/c7sc02664a PMC5868307

[88] Hu W, Fey M, Zitnik M, Dong Y, Ren H, Liu B, Catasta M. Leskovec. J open graph benchmark: Datasets for machine learning on graphs. arXiv. February 2021;24.

[89] Su B, Du D, Yang Z, Zhou Y, Li J, Rao A, Sun H, Lu Z, Wen J-R. A molecular multimodal foundation model associating molecule graphs with natural language. arXiv. September 11, 2022.

[90] Luo Y, Yang K, Hong M, Liu XY, Nie Z. MolFM: A multimodal molecular foundation model. arXiv. July 2023;21.

[91] Sundararajan M, Taly A, Yan Q. Axiomatic attribution for deep networks. Paper presented at: Proceedings of the 34th International Conference on Machine Learning; Sydney, Australia; PMLR; 2017.

[92] Ancona M, Ceolini E, Öztireli C, Gross M. Towards better understanding of gradient-based attribution methods for deep neural networks. Paper presented at: Proceedings of the 35th International Conference on Machine Learning; Stockholm, Sweden; 2018.

[93] Ying Z, Bourgeois D, You J, Zitnik M, Leskovec J. GNNExplainer: Generating explanations for graph neural networks. Paper presented at: Advances in Neural Information Processing Systems; Vancouver, Canada; Curran Associates Inc.; 2019. PMC7138248 32265580

[94] Yu J, Xu T, Rong Y, Bian Y, Huang J, He R. Graph information bottleneck for subgraph recognition. Paper presented at: Proceedings of 10th International Conference on Learning Representations; 2020.

[95] Yu J, Cao J, He R. Improving subgraph recognition with variational graph information bottleneck. Paper presented at: 2022 IEEE/CVF Conference on Computer Vision and Pattern Recognition (CVPR); New Orleans, LA, USA; 2022.

[96] Wu Z, Wang J, Du H, Jiang D, Kang Y, Li D, Pan P, Deng Y, Cao D, Hsieh C-Y, et al. Chemistry-intuitive explanation of graph neural networks for molecular property prediction with substructure masking. Nat Commun. 2023;14(1):2585. 37142585 10.1038/s41467-023-38192-3 PMC10160109

